# Regulation of the Human Ghrelin Promoter Activity by Transcription Factors, NF-*κ*B and Nkx2.2

**DOI:** 10.1155/2015/580908

**Published:** 2015-01-28

**Authors:** Yuki Shiimura, Hideko Ohgusu, Takahiro Sato, Masayasu Kojima

**Affiliations:** Molecular Genetics, Institute of Life Science, Kurume University, Kurume, Fukuoka 839-0864, Japan

## Abstract

To examine the gene expression of ghrelin, a growth hormone releasing and appetite stimulating hormone from stomach, we constructed human ghrelin promoter-reporter vectors and analyzed the promoter activity. The ghrelin promoter activity was high when cultured cells that express ghrelin mRNA endogenously like TT or ECC10 cells were used, indicating that these cells contain factors necessary for full expression of the human ghrelin gene. The human ghrelin promoter contains both positive and negative regulatory regions. A transient decrease of the promoter activity was found when the reporter vector with the −1600 fragment of the human ghrelin promoter was transfected into cultured cells. We then examined the effect of several transcription factors on the ghrelin promoter activity and found that NF-*κ*B suppressed and that Nkx2.2, a homeodomain-containing transcription factor that is important for ghrelin cell development in pancreas, activates the promoter activity. These transcription factors may be possible targets for the control of ghrelin gene expression.

## 1. Introduction

Ghrelin is a peptide hormone that is secreted from stomach and circulates in the blood to activate the release of growth hormone from pituitary and stimulate appetite by acting on hypothalamic arcuate nucleus [1, 2]. Ghrelin is distributed in several tissues, such as gastrointestinal organs, pancreas, and brain, and stomach is the main organ to produce and secrete ghrelin into the circulation [3–6]. The structure of ghrelin is unprecedented; ghrelin is a peptide hormone, in which the serine 3 (Ser3) is* n*-octanoylated and this modification is essential for ghrelin's activity. In the stomach as well as hypothalamus ghrelin exists in two molecular forms; one is* n*-octanoyl modified ghrelin, a major active form. Another is des-acyl ghrelin, which lacks acyl-modification. It is recently suggested that des-acyl ghrelin has different roles from those of* n*-octanoyl ghrelin, although the exact mechanism of the action by des-acyl ghrelin has not been clarified yet [7–10].

Two main physiological functions of ghrelin are growth hormone release and appetite stimulation. Ghrelin dose-dependently stimulates GH release from cultured pituitary cells and injection of ghrelin induces potent GH release in many animal species [11–13]. For the regulation of appetite, when ghrelin was injected into the cerebral ventricles of rats, their food intake was potently stimulated. Moreover, peripherally injected ghrelin also activates hypothalamic neurons in the arcuate nucleus and increases food intake [14–16].

Secretion of ghrelin is controlled by many factors, such as nutrients, peptide hormones, and sympathetic nervous system [17–19]. Moreover, plasma ghrelin level is inversely proportional to BMI [20]. The most important control factor of ghrelin secretion is appetite. Fasting increases expression of ghrelin mRNA and ghrelin peptide production in the stomach and stimulates secretion of ghrelin from stomach [21, 22]. In contrast, food intake decreases mRNA expression and production of ghrelin and suppresses secretion of ghrelin from the stomach [23].

However, the mechanisms of ghrelin mRNA expression and secretion of ghrelin from stomach are not fully understood. In particular, the transcription factor that controls the expression of ghrelin mRNA has not been reported so far, except a possible involvement of upstream stimulatory factor (USF) [24, 25].

We report here that there are positive and negative regulatory regions in the human ghrelin promoter and Nkx2.2 and NF-*κ*B are important regulators for the ghrelin promoter activity. We also determined the several binding sites of Nkx2.2 in the ghrelin promoter.

Recently it is reported that Nkx2.2 determines the fate of pancreatic endocrine cells to differentiate into the beta cell or ghrelin-producing epsilon cell [26]. Our results indicate that Nkx2.2 may function not only in the development of ghrelin producing cells, but also in the regulation of ghrelin gene expression.

## 2. Materials and Methods

### 2.1. Cell Culture

A human gastric carcinoid derived cell line, ECC10 cell, was obtained from RIKEN Cell Bank (Cell number RCB0983) and cultured in RPMI1640 with 10% fetal bovine serum (FBS). TT cell, a human medullary thyroid carcinoma cell line, was cultured in F12-K Nutrient Mixture (Kaighn's Modification, Invitrogen, Carlsbad, CA) with 10% FBS. A monkey kidney cell line, COS-7 cell, was cultured in DMEM with 10% FBS at 37°C in a humidified atmosphere containing 5% CO_2_.

### 2.2. Reverse Transcription and Amplification of Ghrelin cDNA

Total RNAs were prepared from cultured COS-7, TT, ECC10, HEK293, Hep-G2, and AtT-20 cells using RNeasy Mini Kit (QIAGEN, Valencia, CA). The purified RNA sample was incubated in gDNA Wipeout Buffer at 42°C for 2 minutes to remove contaminating genomic DNA. After genomic DNA elimination, the first-strand cDNA was synthesized from 1 *μ*g total RNA using QuantiTect Reverse Transcription (QIAGEN, Valencia, CA) in 1 *μ*L of Quantiscript Reverse Transcriptase, 4 *μ*L of 5x Quantiscript RT Buffer, and 1 *μ*L of RT Primer Mix. The reaction mixture was incubated at 42°C for 15 minutes and then stopped by incubation at 95°C for 3 minutes. PCR was carried out in a 50 *μ*L reaction mixture containing 2 *μ*L of the above first-strand cDNA, 5 *μ*L of 10x PCR buffer, 4 *μ*L of 2.5 mM dNTP mix, 0.3 *μ*M of each primer, and 2.5 U of Taq DNA polymerase (Promega, Madison, WI). RT-PCR primers used were

 human ghrelin forward primer 5′-AAGATGGAGGTCAAGCAGAAGG-3′ and reverse primer 5′-TCCCAGAGGATGTCCTGAAGAA-3′;
 human GAPDH forward primer 5′-GCACCGTCAAGGCTGAGAAC-3′ and reverse primer 5′-ATGGTGGTGAAGACGCCAGT-3′;
 mouse ghrelin forward primer 5′-TTGGCATCAAGCTGTCAGGAG-3′ and reverse primer 5′-GTCAGGCCTGTCCGTGGTTA-3′;
 mouse Rps18 (ribosomal RNA S18) forward primer 5′-AGGATGTGAAGGATGGGAAG-3′ and reverse primer 5′-ACGAAGGCCCCAAAAGTG-3′.

Human GAPDH and mouse ribosomal RNA S18 were used for control. The program used in all samples was 95°C for 2 minutes and then 20 to 35 cycles of 98°C for 20 seconds, 60°C for 30 seconds, and 72°C for 30 seconds. After amplification, PCR products were separated by agarose gel electrophoresis and stained with ethidium bromide. These PCR products were verified by DNA sequencing. We also confirmed that although COS-7 cell is derived from African green monkey kidney cell, the PCR primers used here could amplify the monkey ghrelin cDNA.

### 2.3. Reverse Transcription and Amplification of Transcription Factor cDNA

Total RNAs were prepared from cultured COS-7, TT, and ECC10 cells using RNeasy Mini Kit (QIAGEN, Valencia, CA). The purified RNA sample was incubated in gDNA Wipeout Buffer at 42°C for 2 minutes to remove contaminating genomic DNA. After genomic DNA elimination, the first-strand cDNA was synthesized from 1 *μ*g total RNA using QuantiTect Reverse Transcription (QIAGEN, Valencia, CA) in 1 *μ*L of Quantiscript Reverse Transcriptase, 4 *μ*L of 5x Quantiscript RT Buffer, and 1 *μ*L of RT Primer Mix. The reaction mixture was incubation at 42°C for 15 minutes and then stopped by incubation at 95°C for 3 minutes. PCR was carried out in a 20 *μ*L reaction mixture containing 1 *μ*L of the above first-strand cDNA, 4 *μ*L of 5x PrimeStar buffer, 1.6 *μ*L of 2.5 mM dNTP mix, 0.2 *μ*M of each primer, and 0.5 U of PrimeStar HS DNA polymerase (Takara BIO, Osaka). RT-PCR primers used were

 human NF-*κ*B (p50) forward primer 5′-GCAGATGGCCCATACCTTCA-3′ and reverse primer 5′-CACCATGTCCTTGGGTCCAG-3′;
 human RelA (NF-*κ*B (p65)) forward primer 5′-CCAGACCAACAACAACCCCT-3′ and reverse primer 5′-TCACTCGGCAGATCTTGAGC-3′;
 human Nkx2.2 forward primer 5′-CGCGTGCTTTCAAAGAAGACA-3′ and reverse primer 5′-CACTTGGTCAATTCGTGGCG-3′;
 African green monkey NF-*κ*B (p50) forward primer 5′-CCACCAGGCTTCAGAATGGC-3′ and reverse primer 5′-TTTTCGCTAGAGGCACCAGG-3′;
 African green monkey RelA (NF-*κ*B (p65)) forward primer 5′-GCGAGAGGAGCACAGATACC-3′ and reverse primer 5′-GATGCGCTGAGTGATAGCCT-3′;
 African green monkey Nkx2.2 forward primer 5′-GACTCAAGCTCCAAGTCCCC-3′ and reverse primer 5′-ACCAGATCTTGACCTGCGTG-3′;
 African green monkey GAPDH forward primer 5′-CAGCCTCAAGATCGTCAGCA-3′ and reverse primer 5′-TCTTCTGGGTGGCAGTGATG-3′.

Human GAPDH and African green monkey GAPDH were used for control. The program used in all samples was 98°C for 2 minutes and then 25 to 40 cycles of 98°C for 10 seconds, 55°C for 5 seconds, and 72°C for 20 seconds. After amplification, PCR products were separated by agarose gel electrophoresis and stained with ethidium bromide.

### 2.4. Cloning of the Promoter Region of the Human Ghrelin Gene

The promoter region of the human ghrelin gene was cloned by PCR. The PCR primers used were based on the sequence of the 5′-flanking region of the human ghrelin gene: sense, 5′-AGGATTTAAGAGCGATGGCCGCCTATCTAGAA-3′ (−4000 to −3969) and antisense, 5′-GGCCTCAGCTGGGTTGCAGACAGGTGGGC-3′ (−29 to −1). Human placenta gene library (Clontech, Mountain View, CA) was used as the template. The PCR products were subcloned into the pCRII-TOPO vector (Invitrogen, Carlsbad, CA) and sequenced with a DNA autosequencer (ABI PRISM 3100-Avant Genetic Analyzer, Applied Biosystems, Foster City, CA).

### 2.5. Ghrelin Promoter-Reporter Construction

To analyze the function of the promoter region of the human ghrelin gene, we generated the expression vectors with various deletion mutants of the human ghrelin promoter. Deletion fragments of the human ghrelin gene were generated by PCR with various forward primers starting from −3000, −1996, −1800, −1600, −1400, −1200, −1000, −800, and −605 of the 5′-flanking sequence of the human ghrelin gene. An amplified fragment was subcloned into a reporter plasmid, pGL3 basic vector (Promega, Madison, WI), and fused to the firefly luciferase gene.

### 2.6. Construction of Transcription Factor Expression Plasmids

The Nkx2.2 cDNA (GeneBank Accession number BC075093) was cloned from human pancreas cDNA of MTC Human I cDNA Panel (BD Biosciences, San Jose, CA) by PCR. The NF-*κ*B (p50) cDNA (GeneBank Accession number BC051765) was cloned from human lung cDNA of MTC Human I cDNA Panel (BD Biosciences, San Jose, CA) by PCR. The HindIII and XhoI sites were added to the oligonucleotides and used as the sense and the antisense primers, respectively. PCR products was subcloned into the HindIII-XhoI site of an expression plasmid, pcDNA3.1(+) vector (Invitrogen, Carlsbad, CA). Pax6 (GeneBank Accession number BC011953) and Pax4 (GeneBank Accession number BC074761) cDNAs were cloned from human leukemia cDNA by PCR. ZFP106 (GeneBank Accession number AF205632) and MKRN3 (GeneBank Accession number NM_005664) cDNAs were cloned from human skeletal muscle cDNA (BD Biosciences, San Jose, CA) and human liver cDNA of MTC Human I cDNA Panel (Maxim Biotech, Inc., Rochville, MD), respectively.

### 2.7. Transient Expression Assays of the Reporter Plasmids

TT and ECC10 cells were plated at 1 × 10^6^ cell/well and COS-7 cells were plated at 5 × 10^5^ cell/well in 6-well tissue culture plates (BD Biosciences, San Jose, CA). Cells were maintained in 2 mL of antibiotic-free medium for one day before transfection. Transient transfections were performed using Lipofectamine Plus Reagent (Invitrogen, Carlsbad, CA). In each transfection, 2 *μ*g of the reporter plasmid and 20 ng pRL-CMV were cotransfected into ECC10, TT, or COS-7 cells in 6-well tissue culture plates. After transfection, cells were grown in antibiotic-free medium for 24 hours and plated at 5 × 10^4^ cells/well (TT and ECC10 cells) or 2.5 × 10^4^ cells/well (COS-7 cells) in 96-well tissue culture plate (BD Biosciences, San Jose, CA). Luciferase activity was measured 48 hours after transfection. Luciferase activity was determined in a Fluoroskan Ascent FL (Labsystems, Helsinki, Finland) using the Dual-Luciferase assay system (Promega, Madison, WI) and normalized with the luciferase activity of co-transfected pRL-CMV that contains the cDNA encoding Renilla luciferase (Promega, Madison, WI).

### 2.8. Preparation of Cell Extracts and Electrophoretic Mobility Shift Assay (EMSA)

Nuclear extracts were prepared from TT cells as described previously. EMSAs were conducted using a LightShift Chemiluminescent EMSA Kit (Pierce, Rockford, IL). Four *μ*g of nuclear extract protein was added to the binding buffer containing 10 mM HEPES (pH7.4), 1 mM EDTA, 10% (v/v) glycerol, 100 mM NaCl, 2 mM dithiothreitol, and 1 *μ*g poly (dI/dC). Sequences of the oligonucleotides used in the gel shift assays are shown below:

 −1965 to −1886: 5′-CAG­AGC­TTT­AAA­GTC­TTT­CCA­ACT­CTT­TCA­TTC­TAT­GTC­TCT­CTC­CTC­AAG­AAT­CCT­CAG­TGG­CTG­CCC­TCCT-3′;
 −1631 to −1598: 5′-CAAGCCCCAGCCTAAGGTGAGCTCCTCTCCTGAA-5′;
 −1492 to −1446: 5′-GAAATGAGGCAGTGGCCTTGGCCTATGCTGGGAAGTCCTATG GGCCT-3′;
 −1964 to −1941: 5′-AGAGCTTTAAAGTCTTTCCAACTC-3′;
 −1910 to −1886: 5′-AAGAATCCTCAGTGGCTGCCCTCCT-3′;
 −1492 to −1470: 5′-GAAATGAGGCAGTGGCCTTGGCC-3′;
 −1469 to −1446: 5′-TATGCTGGGAAGTCCTATGGGCCT-3′.

The double-strand oligonucleotide probe was end-labeled using a biotin 3′-end DNA labeling kit (Pierce, Rockford, IL). Binding reactions, which contain 20 fmol of biotinylated probe, were incubated at 4°C for 30 min. The competitor DNA was added at 200-fold molar excess prior to the addition of the probe. Anti-Nkx2.2 antibody (Santa Cruz Biotechnology, Santa Cruz, CA) was preincubated with protein at 4°C for 20 min before adding the probe to the binding reaction. The protein-DNA complexes were resolved on a 5% nondenaturing polyacrylamide gel. After electrophoresis, samples were transferred onto nylon membranes (Hybond-N+, Amersham Biosciences, Piscataway, NJ) and fixed by UV irradiation. After chemiluminescent reactions, the membranes were exposed to X-ray film.

### 2.9. Experimental Animals

C57BL/6JJcl male mice aged 8 weeks were purchased from the CLEA Japan, Inc. (Tokyo, Japan). Mice were housed individually in a temperature-controlled room (24 ± 1°C) under a 12 h light/dark cycle and given ad libitum access to standard laboratory chow and water. The fasted group was deprived of food for 36 h. All procedures were conducted in compliance with protocols approved by the Ethical Committee of the Research of Life Science in Kurume University.

### 2.10. Real-Time PCR of Mouse Transcription Factors

Total RNA (500 ng), extracted from mice stomachs using TRIzol reagent (Invitrogen Tokyo, Japan), was used to synthesize the cDNA templates. Real-time PCR was performed using a StepOne Real-Time PCR System (Applied Biosystems, Foster City, CA). Complementary DNA amplification was performed by using QuantiTect SYBR Green PCR Kit (QIAGEN, Valencia, CA). Each standard well contained the standardized cDNA fragment, which is inserted into the pCII-TOPO vector (Invitrogen Tokyo, Japan). Relative mRNA levels were standardized against a house keeping gene, ribosomal protein S18 (Rps18). RT-PCR primers used were

 mouse NF-*κ*B (p50) forward primer 5′-ACACCTCTGCATATAGCGGC-3′ and reverse primer 5′-GCAGAGTTGTAGCCTCGTGT-3′;
 mouse RelA (NF-*κ*B (p65)) forward primer 5′-GGGCAGTGACGCGACG-3′ and reverse primer 5′-AGCGCCCCTCGCATTTATAG-3′;
 mouse Nkx2.2 forward primer 5′-TGGCCATGTACACGTTCTGA-3′ and reverse primer 5′-CCGATGCTCAGGAGACGAAA-3′.

## 3. Results

### 3.1. Ghrelin mRNA Expression in Cultured Cell Line

**Figure 1:**
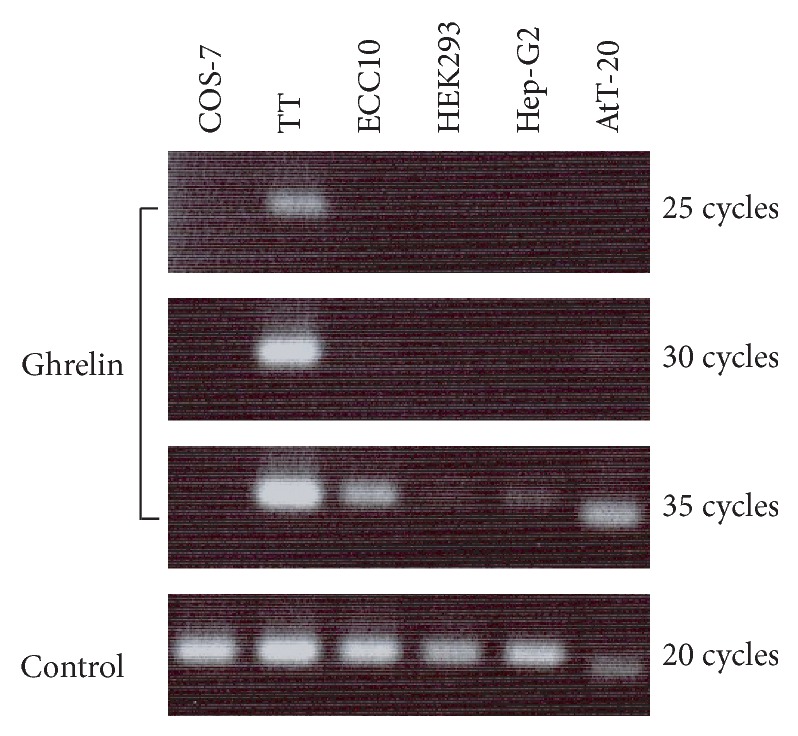
Expression of ghrelin mRNA in cultured cell lines. PCR products were separated by agarose gel electrophoresis and stained with ethidium bromide. Human ghrelin primer set was used for COS-7, TT, ECC10, HEK293, and Hep-G2 cells. Mouse ghrelin primer set was used for AtT20 cell. Controls are human GAPDH for COS-7, TT, ECC10, HEK293, and Hep-G2 cells and mouse ribosomal RNA18 for AtT-20 cell.

To analyze the activity of the human ghrelin promoter, it should be preferable to use a cell line that produces ghrelin endogenously because the cell line seems to contain essential components for the expression of the ghrelin gene. To find a suitable cell line, we screened several cultured cell lines to examine if these cells express ghrelin mRNA endogenously (Figure 1). In the cell lines examined, TT cell, a human thyroid medullary carcinoma cell line, expressed high level of ghrelin mRNA endogenously [27]. PCR cycles of 25 times were sufficient for detecting the amplified cDNA fragment of ghrelin. Other cell lines, COS-7, ECC10, HEK293, Hep-G2, and AtT-20, express lower level of ghrelin mRNA when compared to TT cell. ECC10 and AtT-20 cells need at least 35 cycles of the PCR reaction to detect amplified cDNA fragment of ghrelin. Faint band of the amplified fragment was also observed in HEK293 and Hep-G2 cells after 35 cycles of the PCR reaction. No amplified fragment was observed in COS-7 cell.

**Figure 2:**
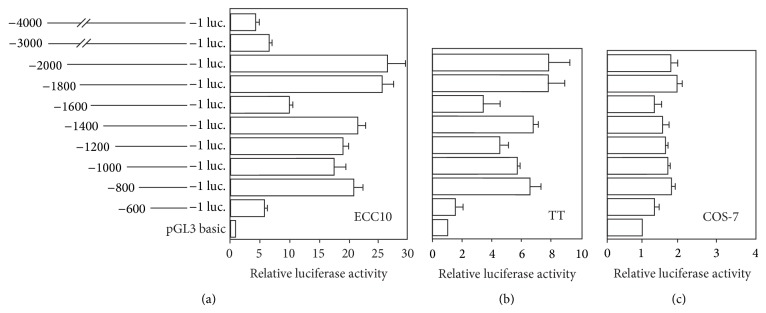
Functional analysis of human ghrelin promoter in cultured cells. Cell specificity of human ghrelin promoter activity was examined. The human ghrelin promoter sequence with different transcription initiation sites (from −4000/+1 to −600/+1) was ligated to the firefly luciferase and inserted into plasmid pGL3 basic. The constructed vectors were transiently transfected into (a) ECC10, (b) TT, and (c) COS-7 cells and the luciferase activities were measured. Relative luciferase activity was the ratio of the luciferase activity cotransfected with each expression vector versus pGL3 basic vector.

Moreover, the activity of the human ghrelin promoter was higher in TT and ECC10 cells than that in COS-7 cells, when the ghrelin promoter/luciferase vectors were transfected into these cell lines (Figure 2). These results indicate that TT and ECC10 cells that endogenously express ghrelin mRNA contain factor(s), which efficiently activates the human ghrelin promoter.

### 3.2. Functional Analysis of the Human Ghrelin Promoter

To identify the regulatory region of the human ghrelin promoter, we constructed several reporter plasmids in pGL3 basic vector that contained various promoter sequences of the human ghrelin gene ligated to a modified firefly luciferase cDNA. Nucleotide position +1 is assigned to the A of the ATG initiator codon of ghrelin and the residues preceding it are indicated by negative numbers. The human ghrelin gene promoter was deleted by every 100~200 bp: the ghrelin promoter fragments used for constructing reporter vectors are −4000/+3, −3000/+3, −2000/+3, −1800/+3, −1700/+3, −1600/+3, −1500/+3, −1400/+3, −1200/+3, −1000/+3, −800/+3, −600/+3, −400/+3, and −200/+3.

The constructed plasmids were transiently transfected into ECC10 cells and the luciferase activity was examined (Figure 2(a)). The luciferase activity was significantly increased when the plasmid that contained the human ghrelin promoter fragment of the −2000/+3 or −1800/+3 was transfected into ECC10 cells. The human ghrelin promoter activity by the plasmid of the −2000/+3 or −1800/+3 fragment was approximately 25-fold higher than that of the promoterless basic plasmid. When the plasmid of the −4000/+3 or −3000/+3 fragment was transfected, the luciferase activity was slightly increased when compared with that of the basic vector. Thus, the region of −2000 to +3 should be the core part of the human ghrelin promoter. Deletion of the promoter gradually decreased the luciferase activity, as reported previously. However, we found transient decrease of the luciferase activity, when the reporter plasmid with the −1600/+3 fragment of the human ghrelin promoter was transfected into ECC10 cells (Figure 2(a)).

The ghrelin promoter activity of the constructed plasmids showed similar pattern when TT cell or COS-7 cell was used for the transfection (Figures 2(b) and 2(c)). Transient decrease of the luciferase activity at −1600/+3 was also observed in both cells. These results indicate that TT and COS-7 cell lines as well as ECC10 cell contain factors that regulate the human ghrelin promoter activity. However, the promoter activity in COS-7 was very low.

We used TT cell for the following experiments because TT cell is easier to treat than ECC10 cell. Moreover, as described above, TT cell endogenously expresses higher ghrelin mRNA than ECC10 cell and produces two forms of ghrelin peptides,* n*-octanoyl modified and des-acyl ghrelins, same as* in vivo* gastric production of ghrelin.

### 3.3. Detailed Analysis of the Transient Suppressive Region of the Ghrelin Promoter

As mentioned in the last section, transient decrease of the luciferase activity was observed when the reporter plasmid inserted with −1600/+3 fragment of the human ghrelin promoter was transfected into ECC10, TT, and COS-7 cells. We used TT cell and examined the region in detail that is responsible for the transient decrease of the promoter activity.

We constructed the reporter plasmids in which DNA fragments derived from various lengths of the human ghrelin promoter between −1805 and −1497 were ligated to the luciferase reporter vector. Then, we deleted DNA fragments of the promoter by every 34 to 36 bp between −1805 and −1497 or 9 to 12 bp between −1771 and −1668.

**Figure 3:**
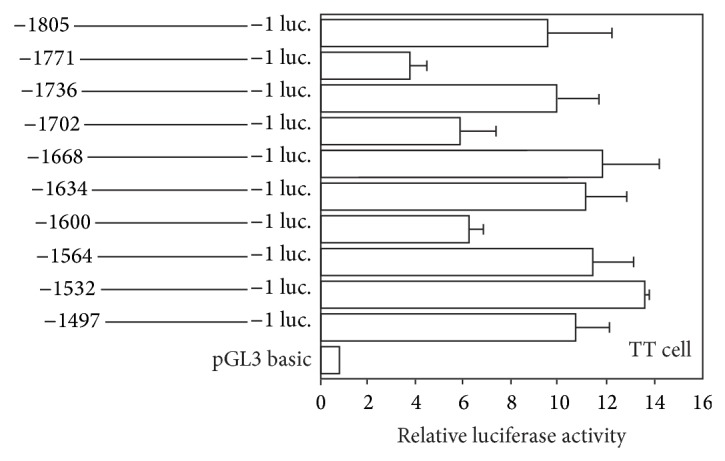
Deletion analysis of the human ghrelin promoter. Relative luciferase activity in the area of −1805 to −1497 of the human ghrelin promoter was examined. Plasmids containing the human ghrelin promoter sequence ligated to the firefly luciferase were transiently transfected into TT cell. The schematic diagram represents the human ghrelin promoter activity in the each deletion constructs. Relative luciferase activity was the ratio of luciferase activity cotransfected with each expression vector versus pGL3 basic vector. The data represent the mean ± SE for triplicate samples. Statistically significant differences were calculated by relative luciferase activity in the area of −1805 to −1 as the standard. Asterisks indicate the differences between each group (*P* < 0.05).

By using these promoter constructs, we found three regions where the luciferase activity was transiently decreased: at −1771, −1702, and −1600, although the latter two regions did not have the statistically significant differences (Figure 3). The responsible regions were very short because additional deletion of only 35~36 bp recovered the decreased luciferase activity.

### 3.4. Binding Sites of Transcription Factors in the Promoter Region of the Human Ghrelin Gene

**Figure 4:**
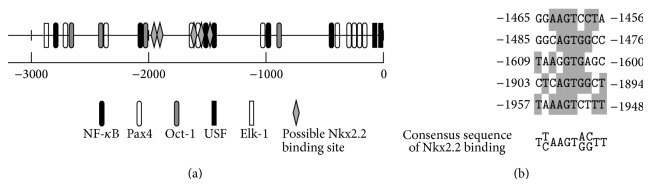
Localization of several transcription factors in the human ghrelin promoter sequence. (a) Possible binding sites of NF-*κ*B, Pax4, Oct-1, USF, Elk-1, and Nkx2.2. (b) Possible Nkx2.2 binding sequences. Nucleic acids that match the consensus Nkx2.2 binding sequence are shaded.

To analyze further the transcription factors that bind to and regulate the ghrelin promoter, we searched possible binding sites of transcription factors. Potential binding sites for various transcription factors were identified by computer-assisted search using AliBaba2.1 (http://www.gene-regulation.com/pub/programs/alibaba2/index.html) and TRANSFAC Match (http://www.gene-regulation.com/cgi-bin/pub/programs/match/bin/match.cgi) programs. As summarized in Figure 4(a), the human ghrelin promoter sequence contained potential binding sites for various transcription factors, such as USF (−63 and −75), NF-*κ*B (−446, −1008, −1464, −1513, −2066, and −2796), Oct-1 (−909, −2045, −2394, and −2666), Elk-1 (−2874), Pax4 (−206, −209, −313, −314, −414, −1010, −1567, −1639, −2366, and −2726), and Pax6 (−414). Moreover, there are five putative binding sites for Nkx2.2 (−1456, −1476, −1600, −1894, and −1948). These Nkx2.2 binding sites do not completely match the representative Nkx2.2 binding site (Figure 4(b)); however, as described below, Nkx2.2 actually binds to some of these sites.

### 3.5. Transactivation of the Human Ghrelin Promoter by Several Transcription Factors

**Figure 5:**
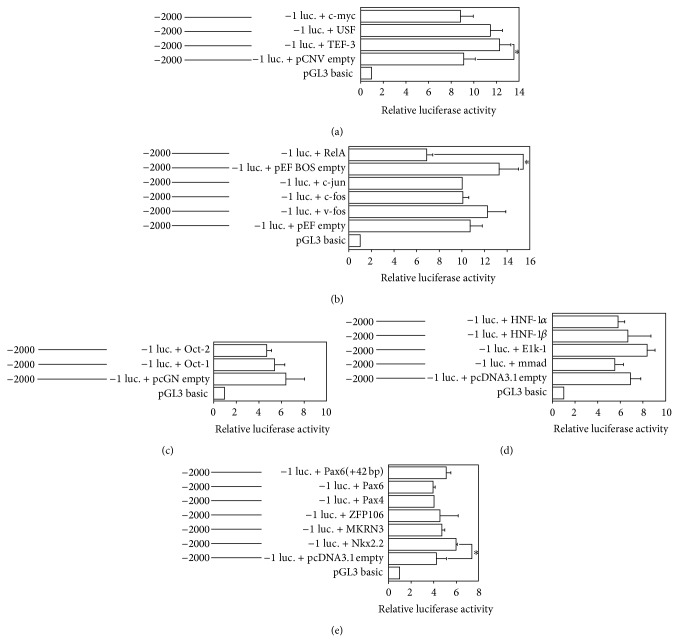
Effects of transcription factors on human ghrelin promoter activity. The vectors used for the experiments are (a) pCNV, (b) pEF-BOS, (c) pcGN, and (d) and (e) pcDNA3.1. The transcription factors are (a) c-myc, USF, and TEF-3, (b) RelA (NF-*κ*B (p65)), c-jun, c-fos, and v-fos, (c) Oct-1, Oct-2, (d) HNF-1*α*, HNF-1*β*, E1k-1, and mmad, and (e) Nkx2.2, Pax6, Pax4, ZFP106, and MKRN3. In transfection experiments, 2 *μ*g of the human ghrelin promoter (−2000/+1), the firefly luciferase reporter plasmid, and 20 ng of pRL-CMV were cotransfected with 25 ng of each transcription factor vector or an empty vector. Promoter activity was normalized by Renilla luciferase activity of the pRL-CMV vector. Relative luciferase activity was expressed as the ratio of luciferase activity cotransfected with each expression vector versus pGL3 basic vector. The data represent the mean ± SE for triplicate samples. Asterisks indicate the differences between each group (*P* < 0.05).

To determine the effects of potential transcription factors, an expression vector of a transcription factor was transfected into TT cells with a human ghrelin promoter reporter plasmid and the luciferase activity was measured (Figures 5(a)–5(e)). We tried several transcription factors, such as USF, RelA (NF-*κ*B (p65)), Oct-1, Elk-1, Pax4, Pax6, and Nkx2.2, because in the human ghrelin promoter sequence the target elements of these factors had been found. We also tried other transcription factors, such as c-myc, TEF-3, c-jun, c-fos, v-fos, HNF1*α*, HNF1*β*, Elk, mmad, Oct-2, ZFP106, and MKRN3. We used the reporter plasmid that was inserted with −2000/+3 of the human ghrelin promoter sequence into pGL3-basic/luciferase vector.

We found that Nkx2.2 and TEF-3 stimulated the promoter activity when the reporter plasmid with −2000/+3 fragment was transfected. On the other hand, RelA (NF-*κ*B (p65)) suppressed the promoter activity when the reporter plasmid with −2000/+3 fragment was transfected. Other factors did not change the promoter activity.

### 3.6. Suppression of the Human Ghrelin Promoter Activity by NF-*κ*B

**Figure 6:**
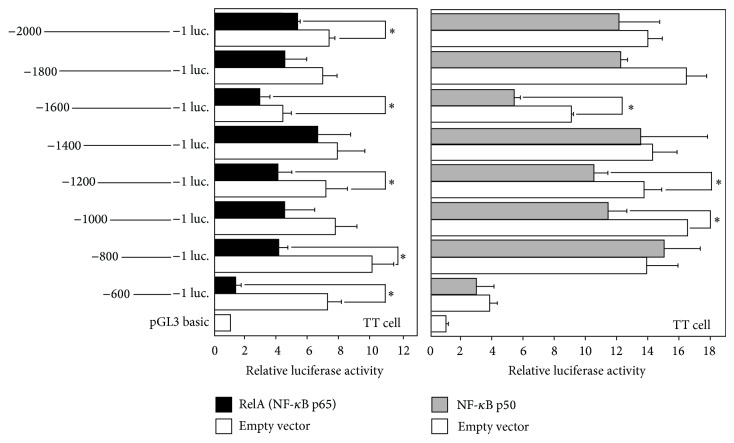
Ghrelin promoter activity by RelA (NF-*κ*B (p65)) and NF-*κ*B (p50). Schematic diagram representing deletion of the human ghrelin promoter introduced into the upstream of the firefly luciferase gene. Each construct (−2000-, −1800-, −1600-, −1400-, −1200-, −1000-, −800-, and −600-Ghrelin-Luc) was transiently cotransfected with pRL-CMV together with each transcription factor vector or an empty vector. Promoter activity was normalized to Renilla luciferase activity. The data represent the mean ± SE for triplicate samples. Asterisks indicate the differences between each group (*P* < 0.05).

From the results of the several transcription factors, RelA (NF-*κ*B (p65)) was shown to suppress ghrelin promoter activity. We then examined which regions of the human ghrelin promoter sequence are important for the suppressive effect by NF-*κ*B family (RelA and NF-*κ*B (p50)) (Figure 6). Figure 6 shows the results of the promoter activities when TT cells were transfected with RelA expressing plasmid and the human ghrelin promoter-luciferase plasmids. The ghrelin promoter activity was significantly suppressed when the reporter plasmids with −800/+3 or −600/+3 promoter regions were transfected. Moreover, suppression of the promoter activity was also observed when the reporter plasmids with −1200/+3, −1600/+3, and −2000/+3 were transfected.

Because there are five members of NF-*κ*B family and these family members form homo- or heterodimer to function in cells, we examined cotransfection of RelA vector and/or NF-*κ*B (p50) expression vector. First, transfection of NF-*κ*B (p50) vector only suppressed the ghrelin promoter when the reporter plasmid with −1600/+3, −1200/+3, or −1000/+3 was transfected (Figure 6). However, no suppressive effect was observed in −800/+3 and −600/+3 promoter regions, which were most potently suppressed when transfected with RelA expressing vector only. Moreover, double transfection of RelA and NF-*κ*B (p50) vector showed similar suppressive pattern to that of NF-*κ*B (p50) only, when −1200/+3 or −2000/+3 promoter plasmid was cotransfected in TT cells. These results suggest that homodimer and heterodimer of NF-*κ*B family differentially regulate the ghrelin promoter activity.

### 3.7. Stimulation of the Human Ghrelin Promoter by Nkx2.2

**Figure 7:**
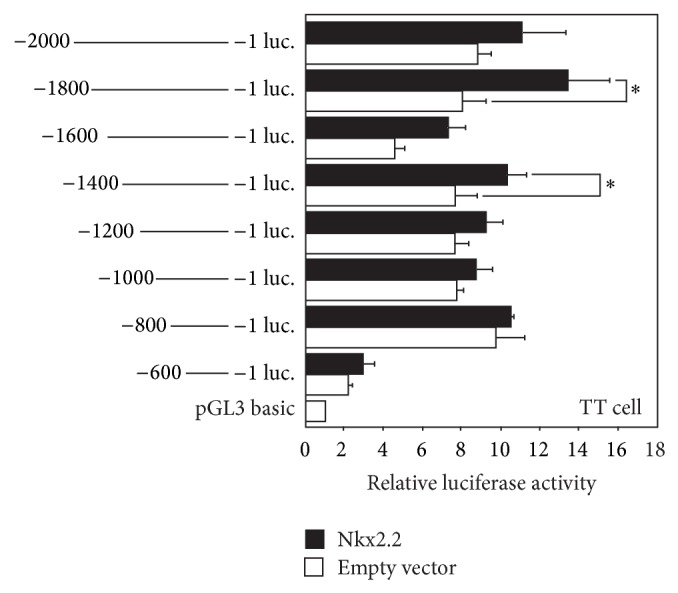
Ghrelin promoter activity by Nkx2.2. Schematic diagram representing deletion of the human ghrelin promoter introduced into the upstream of the firefly luciferase gene. Promoter activity was normalized to Renilla luciferase activity. The data represent the mean ± SE for triplicate samples. Asterisks indicate the differences between each group (*P* < 0.05).

In contrast to NF-*κ*B, which suppresses the promoter activity of the human ghrelin gene, Nkx2.2 stimulates the promoter activity. We then examined which region of the human ghrelin promoter sequence is important for the stimulating effect by Nkx2.2. The ghrelin promoter activity was increased when cells were cotransfected with the Nkx2.2 expressing plasmid and the reporter plasmid, which contained −1800/+3 or −1400/+3 promoter region (Figure 7). These results indicate that the human ghrelin promoter region between −1800 and −1400 is important for Nkx2.2 to stimulate the promoter activity.

### 3.8. Nkx2.2 Binding to the Human Ghrelin Promoter

We searched for possible binding sites of Nkx2.2 in the human ghrelin promoter sequence. Although there are no Nkx2.2 binding sequences that completely match the consensus sequence of the Nkx2.2 binding site in the human ghrelin promoter, we found that there are five possible Nkx2.2 binding sites at −1450, −1483, −1607, −1901, and −1995 (Figure 4) in the human ghrelin promoter sequence. These possible binding sites have similar sequences to the core motif of the binding sites of Nk2 family members.

We next examined whether Nkx2.2 binds to the possible Nkx2.2 binding sites or not. We performed electrophoretic mobility shift assay (EMSA) using synthetic oligonucleotides, which contain the possible binding sites of Nkx2.2 in the human ghrelin promoter sequence, and cell extracts from TT cells. The oligonucleotides used for EMSA were −1492/−1470, −1469/−1446, −1631/−1598, −1910/−1886, and −1964/−1941 of the human ghrelin promoter sequence.

**Figure 8:**
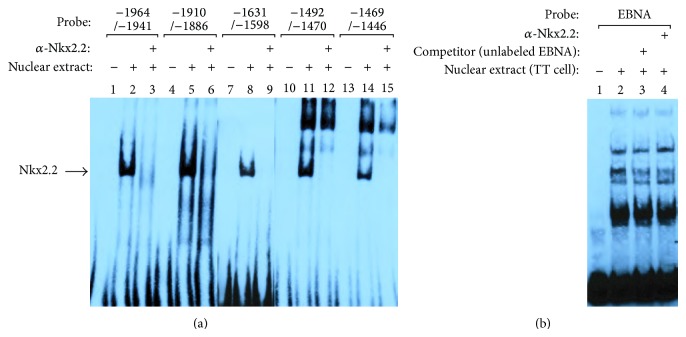
EMSA of Nkx2.2-binding to the human ghrelin gene promoter. (a) Nuclear extracts were prepared from TT cell. The specific complex formed from TT cell nuclear extract and a candidate Nkx2.2 binding element is indicated by an arrowhead. (b) EBNA was used as the control for specific binding of Nkx2.2 antibody. Super shift assay experiments were performed using 1 *μ*g (200 *μ*g/0.1 mL) of Nkx2.2 (H-60) antibody (lanes 3, 6, 9, 12, and 15 in ghrelin promoter assay and lane 4 in control EBNA).

Figure 8 showed the results of EMSA assay. We found that Nkx2.2 actually bound to these sites. Nuclear extracts from TT cells produced a single band of DNA-protein complex in each site (Figure 8(a), lanes 2, 5, 8, 11, and 14). The bands of these complexes were abolished when TT cell extract was preincubated with an Nkx2.2 antibody (Figure 8(a), lanes 3, 6, 9, 12, and 15), which specifically recognizes to the amino acid 214–273 of Nkx2.2 protein.

Control oligonucleotide EBNA also formed several bands of the oligonucleotide and nuclear protein complex when EBNA and the nuclear extracts from TT cells were incubated (Figure 8(b)). However, oligonucleotide-protein complex bands observed in EBNA oligonucleotide were not abolished by unlabeled EBNA oligonucleotide (Figure 8(b), lane 3) or by Nkx2.2 antibody (Figure 8(b), lane 4), indicating that these bands were nonspecific. Thus, oligonucleotide-protein complexes observed in Figure 8(a) were specific for Nkx2.2 and Nkx2.2 specifically binds to the human ghrelin promoter region.

### 3.9. Expression of Transcription Factors in TT, ECC10, and COS-7 Cells

**Figure 9:**
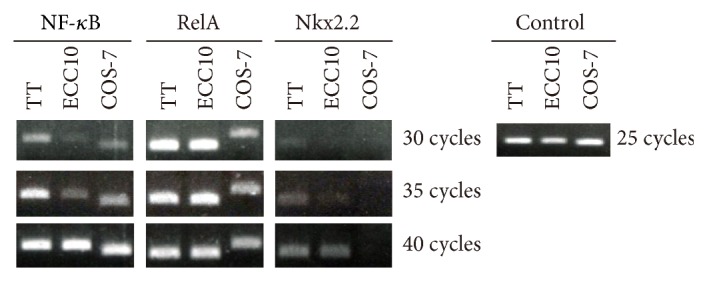
Expression of transcription factors in TT, ECC10, and COS-7 cells. PCR products were separated by agarose gel electrophoresis and stained with ethidium bromide. Human Nkx2.2, NF-*κ*B (NF-*κ*B (p50)), and RelA (NF-*κ*B (p65)) primer sets were used for TT and ECC10 cells. African green monkey Nkx2.2, NF-*κ*B, and RelA primer sets were used for COA-7 cell. Controls were human GAPDH for TT and ECC10 cells and African green monkey GAPDH for COA-7 cell.

Among the three cell lines used in our experiments, higher promoter activity was observed in ECC10 and TT cells. Both cell lines express ghrelin mRNA endogenously. In contrast, COS-7 cell, a cell line that does not express ghrelin mRNA, shows low level of the ghrelin promoter activity. These results indicate that the cell line that endogenously expresses ghrelin mRNA contains essential factor(s) that stimulates the ghrelin promoter activity, while the cell line that does not express ghrelin mRNA lacks factor(s) necessary for full expression of the ghrelin gene. The expression patterns of Nkx2.2, NF-*κ*B (p50), and RelA (NF-*κ*B (p65)) in COS-7, TT, and ECC10 cells support this idea. We found that TT and ECC10 cells expressed Nkx2.2, while COS-7 cell did not express Nkx2.2 (Figure 9). In addition, the three cell lines expressed both NF-*κ*B (p50) and RelA. Because COS-7 does not express ghrelin, these results strongly suggest that Nkx2.2 is an essential factor for ghrelin mRNA expression in culture cells.

### 3.10. Expression of Transcription Factors in Fasting Mice

**Figure 10:**
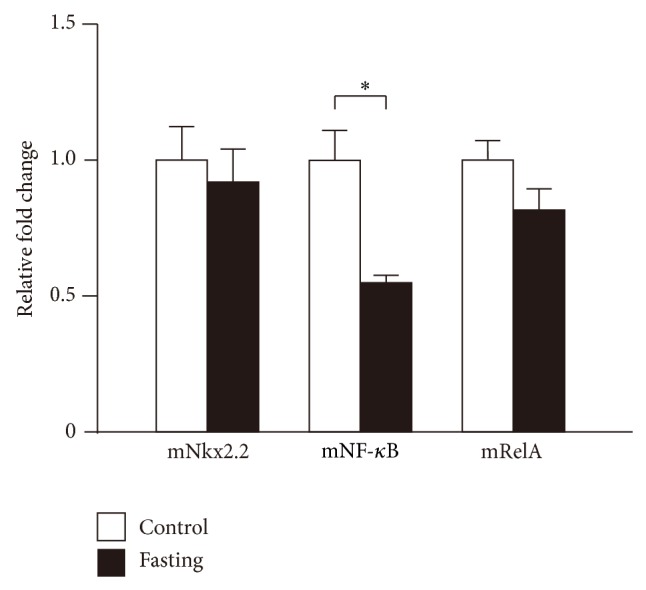
Expression of transcription factors in fasting mice. The cDNA of 36-hour fasting and control mice was synthesized from their stomach mRNAs and used as the templates for PCR analyses. PCR products of mouse Nkx2.2, NF-*κ*B (NF-*κ*B (p50)), and RelA (NF-*κ*B (p65)) were separated by agarose gel electrophoresis and stained with ethidium bromide. Asterisks indicate the differences between each group (*P* < 0.05).

It is well known that fasting induces ghrelin mRNA expression and ghrelin peptide secretion. Then, we examined NF-*κ*B, RelA, and Nkx2.2 mRNA expression levels in the stomachs of fasting mice and found that NF-*κ*B mRNA expression was suppressed, while RelA and Nkx2.2 mRNA expressions showed no change (Figure 10). As shown in this paper, NF-*κ*B suppresses the expression of ghrelin mRNA. Thus, decrease of NF-*κ*B induced by fasting may stimulate the expression of ghrelin mRNA.

## 4. Discussion

In this paper, we examined the promoter activity of the human ghrelin gene and identified several transcription factors that act on the promoter and regulate the expression of the human ghrelin gene. Because ghrelin is a hormone that stimulates growth hormone release and appetite, its regulation is important for controlling metabolic state and body weight of the body [2]. We showed in this paper the following facts on the regulation of ghrelin gene. (1) There are both activating and suppressing regions in the promoter of the human ghrelin gene. (2) Among many transcription factors, Nkx2.2 activates and NF-*κ*B suppresses the ghrelin promoter activity. (3) The binding sites of Nkx2.2 were identified in the promoter sequence of the human ghrelin gene.

The activity of the human ghrelin promoter in this study shows almost similar patterns to those of previous reports [24, 25, 28]. High promoter activity was observed when the reporter plasmid with −2000/+3 to −600/+3 was used for transfection into ECC10 or TT cells. Moreover, deletion of the promoter sequence to less than −300 almost abolished the promoter activity. However, there is no report about the suppressing region in the ghrelin promoter. We found that between −1400 and −1800 of the human ghrelin promoter sequence contained the region that suppresses the promoter activity. This is because we constructed the ghrelin promoter reporter plasmids by every 100 bp deletions of the promoter region and examined the activity in detail. In contrast, other groups did not construct the promoter vectors that the promoter region was deleted by 100 bp like ours.

Deletion of the human ghrelin promoter sequence revealed that the nucleotide between −1400 and −1800 is the negative regulatory region that suppressed the ghrelin promoter activity. Cotransfection of the ghrelin promoter plasmid with −1600/+3 and the plasmid expressing NF-*κ*B (NF-*κ*B (p50) or RelA) suppresses the ghrelin promoter activity. This result indicates that NF-*κ*B may be a factor that is involved in the negative regulation of the ghrelin promoter activity. Moreover, we found that cotransfection of the ghrelin promoter plasmid with −1000/+3 to −600/+3 promoter sequence and the plasmid expressing NF-*κ*B also showed suppressive effects on the ghrelin promoter activity. The region between −1000 and +1 of the human ghrelin promoter contained two possible NF-*κ*B binding sites, although we have not yet succeeded to identify the NF-*κ*B binding sequence that is responsible for suppressing the expression of human ghrelin mRNA.

It is reported that ghrelin and the ghrelin receptor are expressed in T cell and monocyte, indicating the role of ghrelin in immune responses [29–31]. NF-*κ*B is well known to be activated as a central regulator in immune, inflammatory, and stress reactions [32]. NF-*κ*B plays a key role in the immune response induced by lipopolysaccharide (LPS): LPS binds to Toll-like 4 receptor and its downstream signals activate NF-*κ*B [33]. Recent report revealed that LPS injection decreases the plasma ghrelin level, although the mechanism of the ghrelin suppression by LPS has not been clarified [34–36]. We report here that NF-*κ*B suppresses the promoter activity of the human ghrelin gene. Thus, NF-*κ*B may play a key role to suppress the ghrelin level by LPS injection. Moreover, anorexia induced by LPS may be the result of ghrelin suppression through NF-*κ*B activation by LPS.

Ghrelin inhibits both proinflammatory cytokine production and NF-*κ*B activity in human endothelial cells (HUVECs)* in vitro*, and ghrelin suppresses endotoxin-induced cytokine production* in vivo* [37]. These facts strongly suggest that NF-*κ*B is involved in the regulation of an anti-inflammatory action by ghrelin. Thus, ghrelin may be applicable as a possible treatment for inflammation and endotoxin shock.

We examined the effect of Nkx2.2, a homeodomain-containing transcription factor, on the activation of the human ghrelin promoter. The main expression site of Nkx2.2 is pancreas [38], where it is reported that ghrelin mRNA expression is observed and ghrelin peptides are produced in Langerhans islet *α*-cells [4, 39]. Recent reports suggest that mutation of Nkx2.2 causes hyperglycemia [40], a condition that suppresses ghrelin expression and secretion. Moreover, Nkx2.2 is significantly related to development of pancreatic ghrelin cell because lack of Nkx2.2 switches pancreatic progenitor cells to ghrelin-producing epsilon cells [26]. Mice lacking Nkx2.2 have a large number of ghrelin producing cells in pancreas and fail to produce any of the four major islet hormones: insulin, glucagon, somatostatin, and PP (pancreatic polypeptide) [26]. In this paper, we showed that Nkx2.2 activates the human ghrelin promoter and there are several possible Nkx2.2 binding sites in the ghrelin promoter sequence. Although the human ghrelin promoter sequence does not appear to contain complete matched sequences of Nkx2.2 binding sites, we showed that Nkx2.2 actually binds to the possible binding sites in the promoter. Thus, Nkx2.2 is an activator for the human ghrelin promoter as well as a determining factor of the fate of pancreatic cells to develop to ghrelin-producing cells.

Hill et al. reported recently that Nkx2.2 upregulated the ghrelin promoter activity using mouse ghrelin promoter. The promoter regions activated by Nkx2.2 are partially overlapped between human and mouse. Our results for human ghrelin promoter support their findings that Nkx2.2 increases the mRNA expression of ghrelin [41]. Whether overexpression of Nkx2.2 induces obesity or not remains elusive. Thus, Nkx2.2 may be a target molecule to treat feeding disorder through the actions of ghrelin.

Lastly, we examined the promoter region of GOAT, ghrelin O-acyltransferase, and found three candidate binding sites of transcription factors. These are HNF4 at −1950, CDPCR1 at −1379, and Nkx2.5 at −1167. However, there are no common transcription factors between ghrelin and GOAT promoter regions. These results indicate that the regulatory mechanism of GOAT mRNA expression is distinct from that of ghrelin mRNA expression.

## 5. Conclusions

We showed here that the human ghrelin promoter contains both positive and negative regulatory regions. NF-*κ*B suppressed and Nkx2.2 activates the promoter activity. These transcription factors may be possible targets for the control of ghrelin gene expression.
